# A duplicated copy of *id2b* is an unusual sex-determining candidate gene on the Y chromosome of arapaima (*Arapaima gigas*)

**DOI:** 10.1038/s41598-021-01066-z

**Published:** 2021-11-03

**Authors:** Mateus C. Adolfi, Kang Du, Susanne Kneitz, Cédric Cabau, Margot Zahm, Christophe Klopp, Romain Feron, Rômulo V. Paixão, Eduardo S. Varela, Fernanda L. de Almeida, Marcos A. de Oliveira, Rafael H. Nóbrega, Céline Lopez-Roques, Carole Iampietro, Jérôme Lluch, Werner Kloas, Sven Wuertz, Fabian Schaefer, Matthias Stöck, Yann Guiguen, Manfred Schartl

**Affiliations:** 1 Developmental Biochemistry, Biocenter, University of Wuerzburg, Am Hubland, 97074 Wuerzburg, Germany; 2 The Xiphophorus Genetic Stock Center, Department of Chemistry and Biochemistry, Texas State University, San Marcos, Texas, TX 78666 USA; 3 Biochemistry and Cell Biology, Biocenter, University of Wuerzburg, Am Hubland, 97074 Wuerzburg, Germany; 4 Sigenae, GenPhySE, INRAE, ENVT, Université de Toulouse, Castanet Tolosan, France; 5 MIAT, INRA, Université de Toulouse, Chemin de Borde Rouge, 31326 Castanet-Tolosan Cedex, France; 6 INRAE, LPGP, Rennes, France; 7 Department of Ecology and Evolution, University of Lausanne, and Swiss Institute of Bioinformatics, 1015 Lausanne, Switzerland; 8 Embrapa Amazônia Ocidental, Manaus, Amazonas Brazil; 9 Embrapa Pesca E Aquicultura, Palmas, Tocantins Brazil; 10 Reproductive and Molecular Biology Group, Department of Morphology, Institute of Biosciences, UNESP, Botucatu, Brazil; 11 GeT-PlaGe, INRAE, Genotoul, Castanet-Tolosan, France; 12 Leibniz-Institute of Freshwater Ecology and Inland Fisheries, IGB, Müggelseedamm 301 & 310, 12587 Berlin, Germany; 13 Amphibian Research Center, Hiroshima University, Higashi-Hiroshima, 739-8526 Japan; 14 Comprehensive Cancer Center Mainfranken, University Hospital, 97080 Würzburg, Germany

**Keywords:** Evolutionary genetics, Genome, Genetic markers

## Abstract

*Arapaima gigas* is one of the largest freshwater fish species of high ecological and economic importance. Overfishing and habitat destruction are severe threats to the remaining wild populations. By incorporating a chromosomal Hi-C contact map, we improved the arapaima genome assembly to chromosome-level, revealing an unexpected high degree of chromosome rearrangements during evolution of the bonytongues (Osteoglossiformes). Combining this new assembly with pool-sequencing of male and female genomes, we identified *id2bbY*, a duplicated copy of the *inhibitor of DNA binding 2b* (*id2b*) gene on the Y chromosome as candidate male sex-determining gene. A PCR-test for *id2bbY* was developed, demonstrating that this gene is a reliable male-specific marker for genotyping. Expression analyses showed that this gene is expressed in juvenile male gonads. Its paralog, *id2ba*, exhibits a male-biased expression in immature gonads. Transcriptome analyses and protein structure predictions confirm *id2bbY* as a prime candidate for the master sex-determiner. Acting through the TGFβ signaling pathway, *id2bbY* from arapaima would provide the first evidence for a link of this family of transcriptional regulators to sex determination. Our study broadens our current understanding about the evolution of sex determination genetic networks and provide a tool for improving arapaima aquaculture for commercial and conservation purposes.

## Introduction

Sex determination (SD) is the process of commitment of the undifferentiated bipotential gonad to develop towards testis or ovary. In vertebrate, this decision can be triggered by environmental factors (environmental sex determination, ESD), or genetic factors (genotypic sex determination, GSD) or a combination of both^1^. GSD in mammals has a XX/XY chromosome system, and is under control of a single gene, *Sry*, located on the Y chromosome^2^. *Sry* starts to be expressed in XY individuals at a specific time, called the “sex determination window”, activating the male and repressing the female pathway^3^. However, *Sry* is specific for therian mammals, and a high diversity of SD genes is observed in different groups of vertebrates, especially in fish^4,5^. In addition, the complex molecular mechanisms of sex determination are not fully elucidated in mammals and barely understood for other SD genes^6^.

*Arapaima gigas* known in Brazil as pirarucu (“red fish” in the Tupi-Guarani language) is one of the largest freshwater fish on earth. It inhabits the Amazon River and its tributaries. It belongs to the bonytongues (order Osteoglossiformes), an early clade in the phylogenetic tree of teleost fish^7^. Due to its obligatory air breathing and rapid growth, with individuals reaching over 3 m in length and weighting up to 200 kg, research on this species always attracted wide attention^8^. It has high importance for the Amazonian communities’ culture and diet. The scales can be used as nail file, spoon and handicraft, and the bony tongue serves to grate guarana seeds^9^. As a dish this fish is also known as the “Amazon cod”, having a central and important relevance for the economy in the Amazon region, comprising Northern Brazil, Peru, Ecuador, and Colombia^10^. In addition, arapaima is an emerging aquaculture species in South America and most recently also in the USA and Europe. Due to such high demand, arapaima is suffering the negative effects of overfishing, and the reduction of its natural habitats are additionally affecting its survival. Identifying an easily applicable sex marker has been always highly desirable for the arapaima farming industry, allowing precise brood stock formation and consequently increasing the profitability of the sector. At the same time such a tool would be extremely useful for protecting the endangered wild stocks^11^.

Cytogenetic studies demonstrated that the chromosomes of arapaima show homomorphim between male and female, exhibiting 2n = 56, composed of 28 metacentric to submetacentric and 28 subtelocentric to acrocentric chromosomes^12^. Recently, we produced a whole genome sequence of arapaima, and identified that this species has a XX/XY sex chromosome system, meaning that males are heterogametic^13^. In the present study using a chromosomal Hi-C contact map, this assembly has now been improved to chromosome-scale quality. Contrasting genome sequences of gDNA-pools of males and females, we identified the *id2bbY* gene as a reliable male-specific marker in this species. In addition, we performed transcriptome and protein structure analyses, which provided further evidence for *id2bbY* is an excellent candidate as for the male SD gene of arapaima.

## Results

### Genome assembly and annotation

Combining Hi-C sequencing and our previously published scaffold assembly^13^, the contiguity of the genome assembly was improved to chromosome scale. The male and the female arapaima genomes were assembled in 28 large scaffolds each representing the full chromosome complement^14^ and ranging in size from 8 to 45 Mb. Combining gene evidence from homology alignments, expression data mapping and *ab inito* prediction, we annotated 27,439 protein coding genes in the male genome assembly, and 27,379 for the female. The genome annotation improved the BUSCO completeness based on “actinopterygii odb9” to 96%.

### Identification and localization of the male-specific locus of arapaima

**Figure 1:**
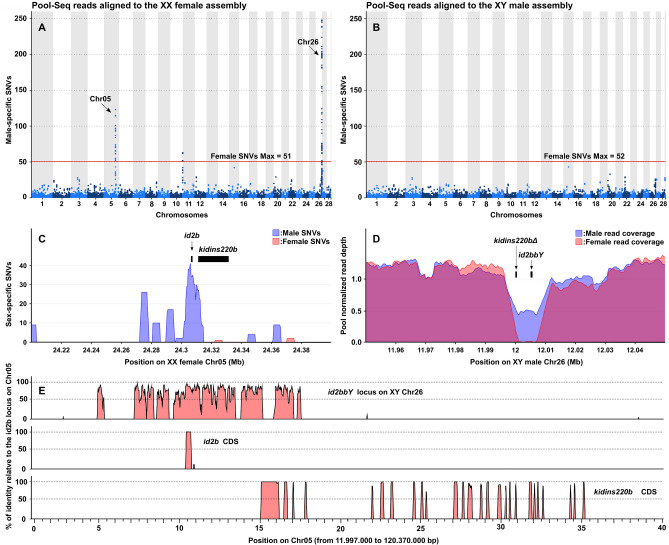
Characterization of the sex chromosome and sex-locus of *Arapaima gigas*. (**A, B**) Genome-wide Manhattan plot visualization of male-specific single-nucleotide polymorphisms (SNVs) along the 28 chromosomes (Chr) of the female (**A**) and male (**B**) *A. gigas* genome assemblies. Male-specific SNVs are represented as dots (total per 50 kb window size) of alternating colors to distinguish their location on adjacent chromosomes. The maximum genome-wide values of female-specific SNVs are shown on the plots (red lines). (**C**) Zoomed view of the sex-specific SNVs (total per 5 kb window size) on the sex-biased region of Chr05 with the location of the inhibitor of DNA binding 2 b gene (*id2b*) and of the kinase D interacting substrate 220 b gene (*kidins220b*). (**D**) Zoomed view of the normalized coverage depth (average per 5 kb window size) of the sex-biased Chr05 homologous region on male Chr26 with location of the duplicated *id2bbY* copy of the Chr05 *id2b* gene and of the truncated duplicated *kidins220bΔ* of the Chr05 *kidins220b*.E. Multiple alignment plots of the percentage (%) of sequence identity between the *id2bbY* locus on Chr26, the coding sequences (CDS) of *id2b* and *kidins220b* and the corresponding autosomal Chr05 region used as a reference.

To identify the sex locus of arapaima, we sequenced a pool of male and a pool of female genomic DNAs. These pool-sequencing (Pool-Seq) datasets were aligned to the male (heterogametic sex, XY) and female (homogametic sex, XX) assemblies to search for sex-biased signatures. When the Pool-Seq reads were aligned to the female assembly, two prominent peaks of male-specific single nucleotide variants (SNVs) were detected (heterozygous variations in males but homozygous for the same allele in all females) on chromosomes (Chr) 26 and 5, and a minor peak on Chr 10 (Fig. 1A). No signal of sex-linkage appeared using the male reference assembly, however (Fig. 1B). The sequence containing the peak of male-specific SNVs in the female assembly Chr05 (Fig. 1C) has high sequence identity with a small region of the male assembly of Chr26 (Fig. 1E). In the male pool, the latter region’s normalized read coverage is half that of sequences elsewhere on the chromosome, and a 10 kb region has a complete absence of reads in the female pool (Fig. 1D). These results suggest that the distal part of Chr26 contains the sex locus of arapaima and that this originated by insertion of a small duplicated fragment region from a progenitor on the autosomal Chr05. The high male-specific SNV-peak detected on the female Chr26, may therefore result from a larger X/Y differentiated region around the small male sex-specific insertion, since, in general, the sex-determining region is known to accumulate mutations around the SD gene^15^. As the arapaima sex determination system is XX/XY male heterogamety^13^, the lack of signal on the male assembly could be considered as counter-intuitive. However, our findings are consistent with a male-specific duplication/insertion, whose male-specific reads can only align to the autosomal region of the XX female genome from which this duplication originated. Hence, the Y-specific sequences are haploid and no male-specific SNVs are expected in those regions, as females have no corresponding sequences in their genome to be compared with. This provides a clear and strong signal on that location on Chr05, which is easier to detect than the small region with coverage difference between the X and Y versions Chr26.

### A duplicated *id2b* gene in the male-specific region of the Y chromosome

This small male-specific region (9656 bp) on Chr26, which shows high similarity to Chr05 (Fig. 1D), contains only two genes, a duplicated copy of *id2b* (*id2bbY,* [535 bp (DNA) and 411 bp (CDS)]) and a fragment of *kidins220b* (*kidins220bΔ*, [514 bp (DNA) and 231 bp (CDS)]) (Fig. 1D). The DNA sequence of *kidins220bΔ* is highly diverged (5.7% amino acid and 6.1% cDNA identity) from to the homologous *kidins220b* (15,043 bp DNA and 3477 bp CDS) on Chr05 suggesting that this gene fragment is corrupted. All RNA-seq datasets examined (see “Methods”) lacked transcripts from the Chr26, supporting the view that it is non-functional. In contrast, the *id2bbY* copy is highly conserved, with 93.2% cDNA identity and 86.9% amino acid identity.

**Figure 2:**
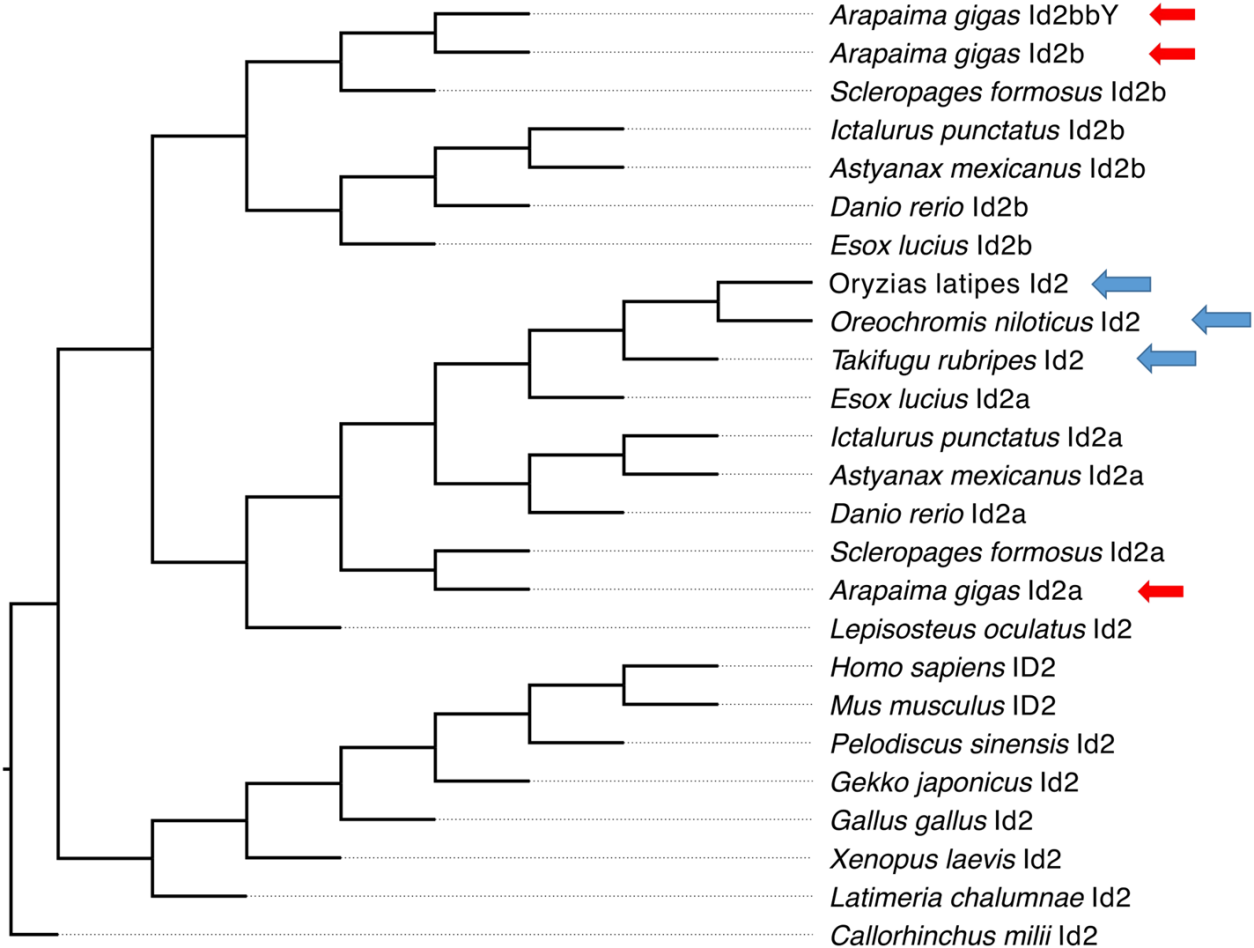
Phylogenetic analyses of Id2 (Id2a) and Id2b and Id2bbY amino acid sequences in vertebrates. The topology of the gene tree follows the expected phylogenetic relationships of the organisms. Red arrows show the Id2 sequences in arapaima. Blue arrows indicate the species that lost the *id2b* gene.

Due to the third round (3R) of whole-genome duplication (WGD) of teleosts, many genes known from other vertebrates exist as in two versions, so-called ohnologs. Our analyses of the arapaima genomes identified two copies of *id2* in females according to the 3R expectation. However, three copies were observed in males. To reconstruct the evolution of the *id2* gene family in vertebrates, were retrieved all genes with annotation “id2” from the fully sequenced high-quality reference genomes of 9 teleost species, spotted gar (basal actinopterygian), human, mouse, chicken, turtle, gecko, African clawed frog, coelacanth, and elephant shark to build a gene tree (Fig. 2). Phylogenetic analyses revealed two ohnologs of *id2*, *id2a* and *id2b*, derived from the 3R teleost specific WGD. The male-specific gene, *id2bbY*, was only retrieved from arapaima and is a Y-chromosomal paralogue of the autosomal *id2b*, named *id2ba* (Fig. 2).

Of note, *id2a* has been retained in all analyzed teleost species, while *id2b* was lost in more derived groups of teleosts, such as medaka, tilapia and fugu, and all other Percomorpha (http://www.ensembl.org/Homo_sapiens/Gene/Compara_Tree?db=core;g=ENSG00000115738;r=2:8678845-8684461;collapse). After a duplication, the loss of one out of two gene copies is expected to be linked to relaxation of purifying selection. To investigate the situation for the ohnologous gene pair *id2a* and *id2b* after their divergence, we investigated the ω (= Ka/Ks) values. With all *id2b* genes set to one ω value and the *id2*/*id2a* genes to a different one in PAML, we found that the two-ratio branch model fits significantly better than the one-ratio model (lnL − 3514.921109 vs. − 3535.819830, *p* value < 0.01, likelihood ratio test), and the ω value of *id2b* (0.21) is two times that of the other *id2* genes (0.10) (Fig. S1). This indicates a relaxation of purifying selection on *id2b* after its divergence from *id2a*, which may be connected to the multiple losses of *id2b* during evolution.

To assess whether *id2bbY* emerged before or after the divergence of arapaima and Asian arowana, we screened the whole genome assembly of arowana for *id2bbY* orthologs but failed to detect it. Thus, *id2bbY* either arose before the split of arapaima and arowana but was subsequently lost in arowana, or the gene duplication event occurred after the divergence of both fish lineages. To answer this question, we compared the Ks distance between arapaima *id2bbY* and *id2ba* to the genome-wide Ks between arapaima and arowana. The Ks distance represents synonymous changes, which are generally not exposed to selection. Hence, this parameter can be used under the assumption of an appropriate neutral molecular clock. We retrieved 18,621 one to one orthologous pairs with conserved synteny between arapaima and arowana. Their pairwise Ks values were then estimated and resulted in a distribution with median at 0.44 and mean at 0.46, while the Ks distance between arapaima *id2bbY* and *id2ba* was estimated to be 0.078, which is far smaller. This indicates that *id2bbY* emerged after arapaima diverged from arowana, and hence is a specific duplication in the arapaima lineage. Assuming the divergence time between arapaima and Asian arowana at 106 MYA^16^ and the Ks at 0.46, *id2bbY* emerged around 18 MYA.

### The *id2bbY* is a male-specific marker of arapaima

To validate if *id2bbY* can be used as a male-specific marker in arapaima, PCR amplifications with specific primers were performed in 8 different populations (Table 1). In our previous study, 25 males and 25 females derived from two different populations (Senador Guiomard and Cacoal, Brazil), were used for RAD-tag analyses^13^. Male and female sex-specific RAD-tags were extracted from those animals. While generally a good match between genotyped and phenotyped was recorded, three males showed a female pattern of tags (males #10, #13 and #24), and one female showed male tags (female #21). We genotyped all 50 animals for the *id2bbY* gene. Results fully agreed with RAD-tag genotyping and we confirmed that the outliers belong to the opposite sex (Fig. S2 A and B). Most likely these outlier individuals were not accurately phenotypically sexed, which was done in the fish farms by gross body morphology. Similarly, the population from Pentecoste showed 3 outliers, showing the difficulties in identifying the sex of arapaima either by endoscopy of the gonad or by vitellogenin detection, which accurately identifies mature (vitellogenin producing) female, but with a risk to mis-classify immature females (non-vitellogenin producing if not induced by 17β-estradiol at this reproductive stage)^17^ as males. Accordingly, all animals that were sexed using histology, a much more precise procedure, showed 100% concordance of phenotypic sex with the *id2bbY* genotype.

**Table 1:** Comparison between different morphological sexing procedures and PCR efficiency for *id2bbY* gene.

Population	Phenotype				Accuracy (%)	Sexing procedure
	Males		Females			
	Total number	Confirmed	Total number	Confirmed		
Cacoal—RO	8	7	7	7	93.33	Gross body morphology
Coari—AM	4	4	7	7	100.00	Gonad histology
Pentecoste—CE	16	14	13	12	89.66	Gonad endoscopy or vitellogenin detection
Pimenta Bueno—RO	8	8	4	4	100.00	Gonad histology
Presidente Figueiredo—AM	6	6	4	4	100.00	Gonad histology
Senador Guiomard—AC	17	15	18	17	91.43	Gross body morphology
Senador La Rocque—MA	13	13	9	9	100.00	Gonad histology
Thuringia	11	11	13	13	100.00	Gonad histology
Total	83	78	75	73	95.57	

The total numbers are the animals sexed by morphology, and the confirmed numbers are those that genotypically match to the expected sex.

### *id2bbY* is a candidate master sex-determining gene in arapaima

The origin of the *id2bbY* gene resembles the situation in medaka, in which an autosomal gene was duplicated and the new copy inserted as a small male-specific region into another chromosome, thus generating the proto-Y^18^. In medaka, the SD gene *dmrt1bY* (synonym *dmy*) is evolving faster than its ancestral gene, *dmrt1a*^19^, which supports of the hypothesis of a higher mutation rate in males than females, due to greater number of cell divisions in the male germ line^20^ and/or lower copy number of Y chromosome compared to X chromosomes and autosomes, allowing genetic drift to act more strongly (collectively named “Y-driven evolution”). To assess if this is also the case for the arapaima candidate SD gene, we estimated the substitution rates across the gene tree of *id2* (including *id2a*, *id2b* and *id2bbY*) using codeml under the free-ratio model. This analysis revealed that the synonymous substitution rate (Ks) of the arapaima *id2bbY* branch (0.0888) is 14 times higher than of the arapaima *id2b* branch (0.0062). In addition, the mutation rate between the intronic region of *id2bbY* and *id2b* was calculated to exclude the possibility that the high Ks observed for the *id2bbY* branch is due to a codon usage bias. Using the arowana *id2b* as the reference, the pairwise distance (*p*-distance model in MEGA) of arapaima *id2bbY* intron (0.427) is longer than that of the arapaima *id2b* intron (0.387), consistent with the hypothesis of Y-driven evolution.

In medaka, *dmrt1bY* has a higher ω value compared to its ancestor *dmrt1a*^19^. Similarly, *id2bbY* shows a higher ω value (0.97) than *id2b* (0.25, Fig. S3). This is estimated under a model^21^ of one ω for *id2bbY* and a different one for *id2ba*. Among all branches, the log likelihood value under this model (− 1721.39) is higher than that (− 1724.89) of the model with an equal ω value but misses marginally the significance level (*p* = 0.06, likelihood ratio test). In spite of the higher ω, using the branch-site model^21^, we failed to detect positively selected sites in *id2bbY*.

**Figure 3:**
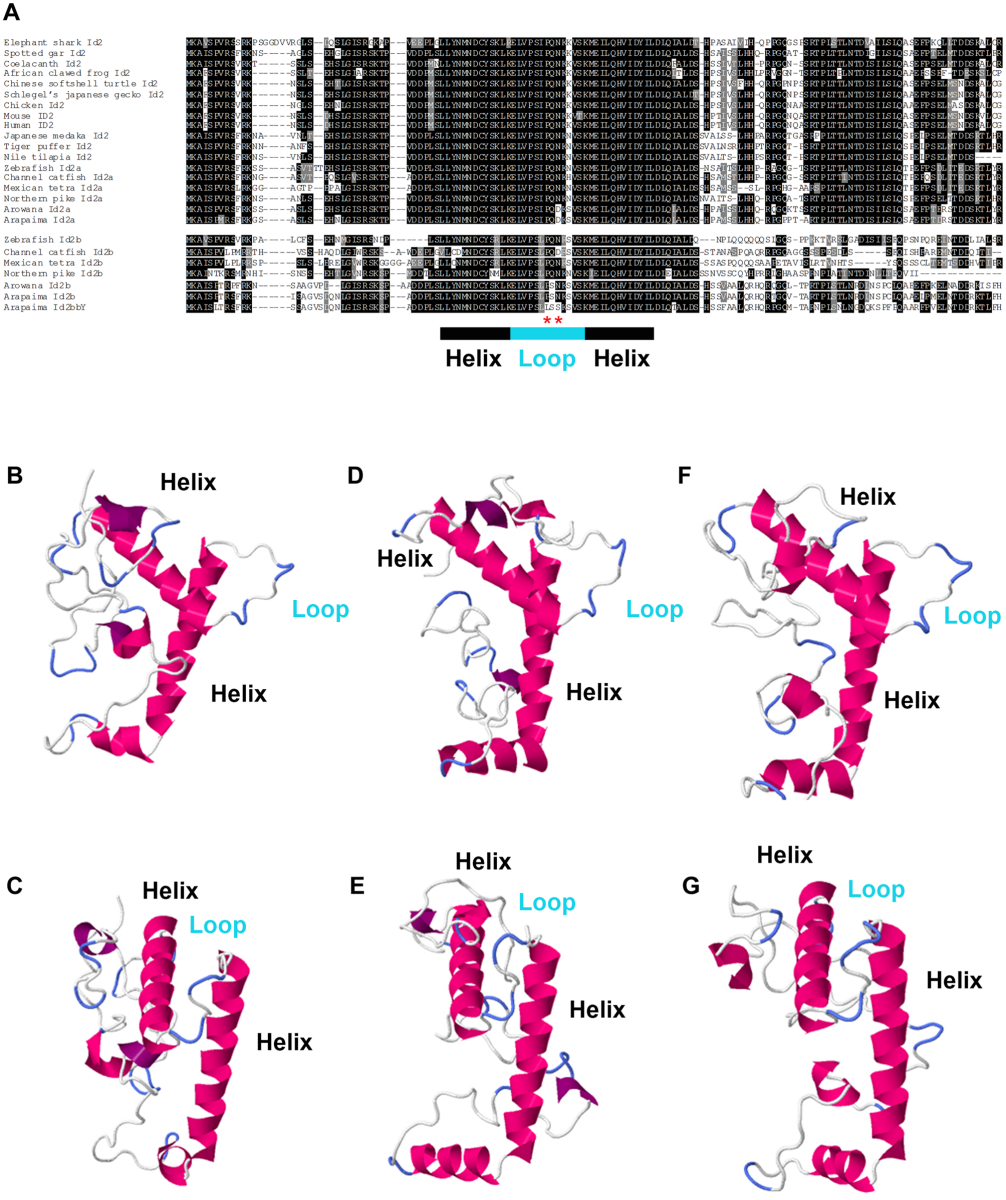
Primary and tertiary structure of Id2 proteins. (**A**) Amino acid sequence alignment of Id2 in vertebrates and localization of the HLH domain. Red stars display the amino acid changes exclusive for Id2bbY. Structure prediction of Id2a (**B**, **C**), Id2ba (**D**, **E**) and Id2bbY (**F**, **G**) of arapaima.

To provide further evidence that *id2bbY* is most likely the male sex-determining gene of arapaima, we performed protein structure and expression analyses of the *id2* genes. The Id2 proteins belong to the inhibitor of DNA binding (ID) family, which is characterized by a helix-loop-helix (HLH) domain. The ID family proteins do not bind to DNA, instead they interact directly with basic helix–loop–helix (bHLH) transcription factors, suppressing their heterodimerization and inhibiting their action in a dominant-negative manner. The amino acid sequence comparison between the Id2’s of different vertebrate species revealed that Id2a is more conserved than Id2b (Fig. 3A). The divergence of Id2b sequences is also observed in the HLH domains of catfish, Mexican tetra, and Northern pike. However, in zebrafish, arowana and arapaima, the HLH domain of the Id2b’s is similar to that of Id2a. Interestingly, besides changes at more variable positions, the Id2bbY peptide sequence has a proline to leucine (P56L) and an asparagine to serine (N58S) amino acid replacement in the highly conserved loop of its HLH domain. Protein structure prediction of Id2a, Id2ba and Id2bbY in arapaima revealed no obvious change in the 3-D structure of Id2bbY, indicating that it is a functional Id2 factor. These results motivate in-depth structure/function relationship studies in the future (Fig. 3B–G).

**Figure 4:**
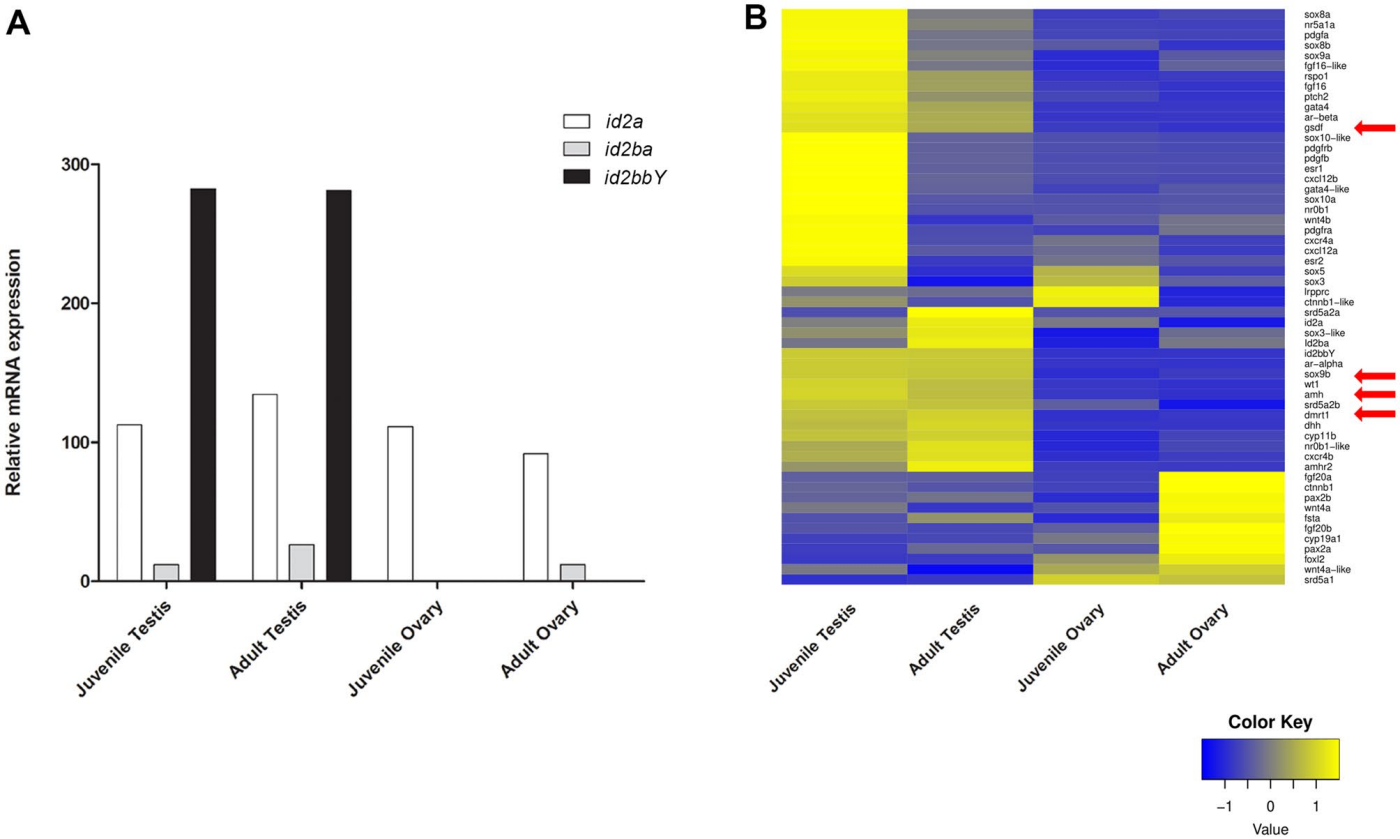
Transcriptome analyses of sex-related genes in juvenile and adult gonads of male and female. (**A**) Relative mRNA expression comparison between *id2a*, *id2ba* and *id2bbY*. (**B**) Heat map of sex-related genes showing higher expression levels in yellow and lower expression in blue. Arrows indicate transcription factors and growth factor genes important for testis development.

Sex-determining genes are expected to show an expression bias towards one of the sexes during early gonadal development. In addition, Y-specific SD genes (e.g. *gsdf*^*Y*^*, gdf6Y*, *amhy*), derived by gene duplications or allelic variation display higher expression levels in the gonads during the early stages of sex determination and sex differentiation when compared to the autosomal copy or to the version on the X chromosome, respectively. For expression analyses, first two juvenile arapaima (about 1 year old and between 94 and 102 cm long) from the Thuringia aquaculture were used for RNA-seq. At this stage, females showed ovaries containing mainly oogonia and pre-vitellogenic oocytes, while male testis tubules were characterized by germinal epithelium containing only Sertoli cells and spermatogonia (Fig. S2C, D). Expression analyses of *id2a*, *id2ba* and *id2bbY* revealed that both, *id2ba* and *id2bbY*, exhibit male-specific expression in juveniles, with *id2bbY* being almost 10 times more expressed than *id2ba* (Fig. 4A). In previously established transcriptomes of adult gonads^13^, *id2bbY* expression is similar to the juvenile gonads. However, *id2ba* is upregulated in adult testis and ovary compared to the juvenile stages of the organs (Fig. 4A). The expression levels of *id2a* are similar in all samples, being slightly lower in adult ovaries (Fig. 4A). In summary, *id2bbY* follows the expected expression pattern of a potential sex-determining gene.

Transcriptome analyses of genes known to be involved in sex determination and gonadal differentiation in vertebrates showed that the male and female genes are active in both juvenile testis and ovary (Fig. 4B). Transcription factors important for testis development, e.g., *dmrt1* and *sox9b,* showed similarly high expression in juvenile and adult males. In addition, growth factors related to TGFβ signaling, *gsdf* and *amh,* which are important for testis differentiation, showed higher expression in juvenile testis than in the adult (Fig. 4B).

## Discussion

Our high-quality chromosome-level genome of a male and a female arapaima considerably improved the former published genomic information. The new assemblies allowed to identify a most likely candidate for the male sex-determining gene and to establish a highly versatile PCR test for sex genotyping.

Sex chromosome evolution can be a relatively rapid process, and it depends on the origin and fixation of a new SD gene^22^. Both Asian arowana and arapaima belong to the suborder Osteoglossoidei. While arapaima has a XX/XY system, Asian arowana females possess heteromorphic sex chromosomes, indicating a ZZ/ZW system^23^. A similar situation has been described for fish of the genus *Oryzias*, which further demonstrates that SD genes and sex chromosome systems can vary even between closely related species. The *dmrt1bY* gene (on LG1) is the SD gene in both *Oryzias latipes*^24,25^ and *Oryzias curvinotus*^26^, while *gsdf*^*Y*^ (LG12) is the SD gene in *Oryzias luzonensis*^27^ and *sox3*^*Y*^ (LG10) in *Oryzias dancena*^28^. *Oryzias hubbsi* and *Oryzias javanicus,* differently from the other species of the genus, even have a ZZ/ZW sex chromosome system^29^.

**Figure 5:**
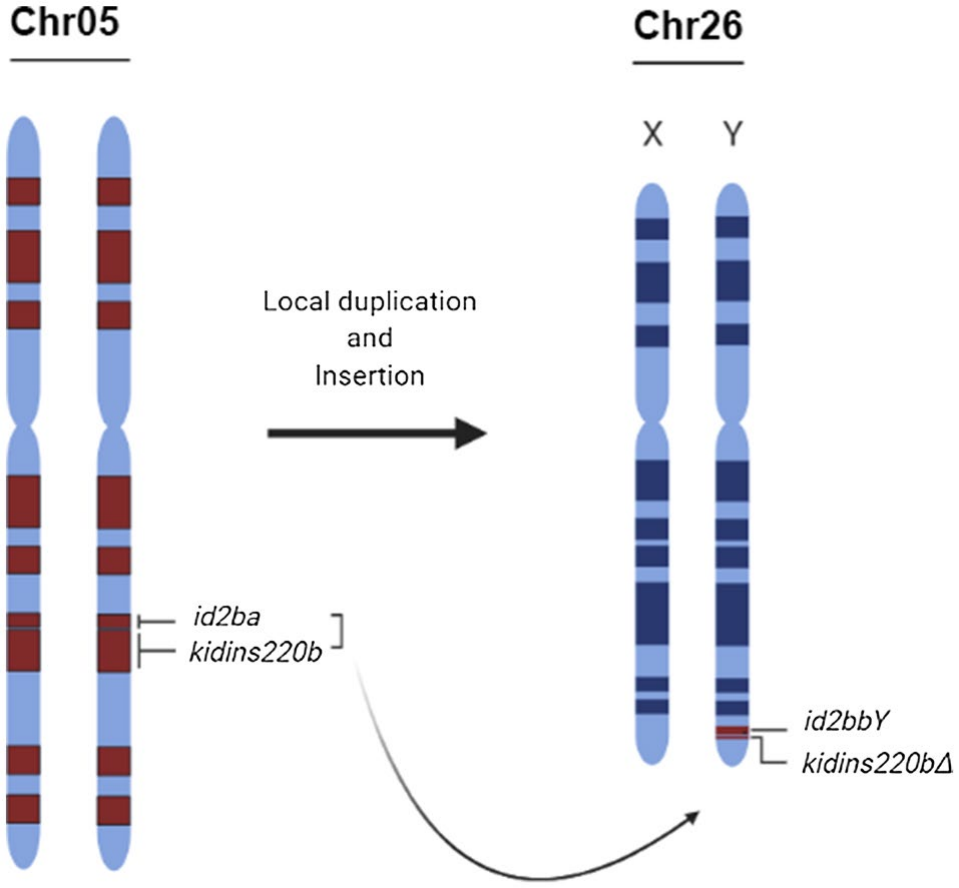
Evolutionary origin of the arapaima Y-chromosome. Schematic representation of a local DNA duplication on chromosome (Chr) 5 followed by an insertion of the duplicated fragment into Chr26 (sex chromosomes) of arapaima. The duplication contains the male-specific marker *id2bbY* and a truncated copy of *kidins220b* gene, *kidins220bΔ*. Created in BioRender.com.

The *id2bbY* gene is a duplicated copy of the autosome *id2b* gene on the Y chromosome (Fig. 5). Sequence differences from the autosomal precursor make it possible to use this gene as male-specific molecular marker. The importance of finding a marker is extremely useful for sex ratio control in aquaculture, for instance for the production of monosex populations, and for monitoring wild populations. These are desirable because of the existence of valuable traits associated with one sex (e.g. growth, color and shape)^11^. In arapaima, the giant size, and the long time to reach sexual maturation require a huge amount of resources to maintain the animals for the breeding process ^9^. The most accurate method to identify the sex of an individual is the direct inspection of the differentiated gonad morphology confirmed by histology, especially for immature fish. However, for rearing purposes, other methods have to be applied, such as endoscopy and vitellogenin detection. The simple PCR amplification of *id2bbY* showed 100% accuracy in reliably sexed fish. This method is minimally invasive, and requires only a very small fin clip or oral swap and can be done already in small fish. This genotyping method was validated in different populations of arapaima, distributed along the Amazon territory (Fig. S4). Despite the broad range, recent studies have provided no evidence for the existence of different species of arapaima, and that the genetic divergence between populations is associated with sedentary behavior, the impact of fisheries on stocks, and the characteristics of each basin, e.g., floodplain dynamics^30–33^.

**Figure 6:**
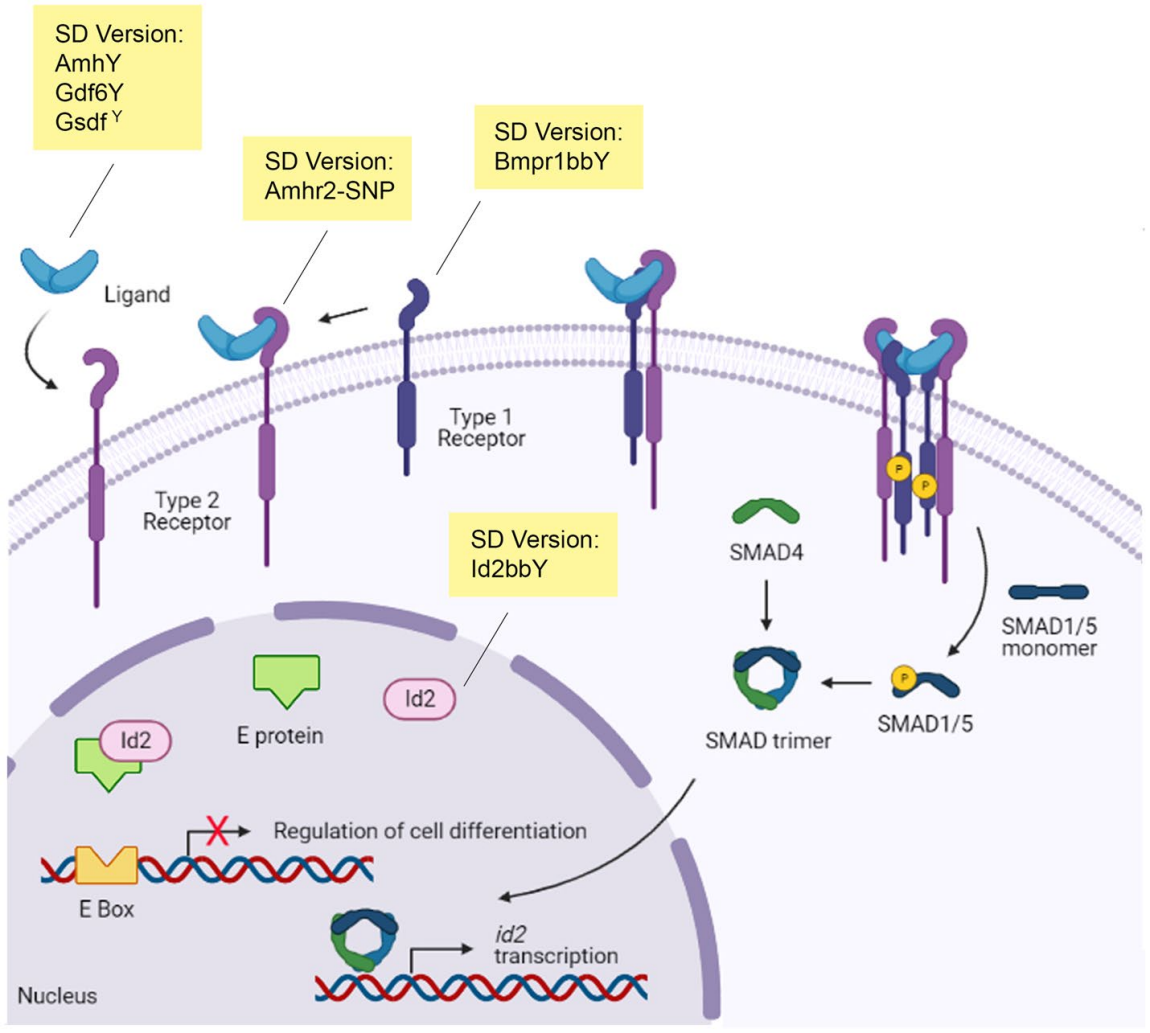
Conceptual links between TGF-β signaling, *id2* and known sex-determining genes. Ligands belonging to the TGF-β superfamily bind to their respective type 2 receptor, which in turn recruits the type 1 receptor activating the SMAD factors and leading to expression of *id2*. The Id2 protein interacts directly with E proteins therefore preventing their binding to DNA in the E Box sequence. Different components of the TGF-β signaling pathway recurrently became the sex-determining gene, where Id2b would be in a downstream position in the pathway. Created in BioRender.com.

All SD genes described so far are single gene duplicates or allelic variants of genes known to be related to sex determination and differentiation^34^. The only exception is the *sdY* gene in salmonids, a truncated copy of the *irf9* (interferon regulatory factor 9) gene^35^, which has not been implicated in sexual development so far. At first sight, *id2bbY* appears as another exception because to date, *id2* has never been assigned an important role in sex determination and differentiation. However, several studies showed that this gene and other ID proteins are involved in ovary maturation, granulosa cell differentiation and spermatogenesis^36–38^. In chicken, Id2 was proposed to be involved in ovarian follicle differentiation by increasing the levels of *fshr* mRNA^39^. The Id genes are transcriptionally regulated by TGF-β signaling^40^, which is known to be important for ovary and testis development, and germ cell differentiation^41,42^. TGF-β growth factors bind to their respective type 2 receptors, which in turn recruit a type 1 receptor activating the SMAD factors that regulate *id2* gene expression^43^ (Fig. 6). Components of the TGF-β signaling pathway recurrently became the SD trigger in different species, including *amhy*^44^, *gdf6Y*^45^, *gsdf*^*Y*^^27^, *bmpr1bbY*^46^ and *amhr2-SNP*^47^. Interestingly, those SD genes encode ligands and receptors, and therefore are located upstream in the signaling pathway. The *id2bbY* of arapaima would be the first reported example of a downstream factor of the TGF-β cascade that has evolved as master male SD gene (Fig. 6). A sex-linked SNP mutation in *amhr2* of obscure puffer (*Takifugu obscurus*) showed that males have higher phosphorylation levels of Smads and also higher activity of *id3* when compared to females^48^. Recent data using single-cell sequencing demonstrated that, in fetal testis of humans, *ID2* upregulation is necessary for germ cell and testis development, and that it is induced by *AMH* and *BMPR1B*^49^. The current knowledge suggests that an increase of TGF-β signaling during the sex determining window, in this case caused by *id2*, can lead to testis development.

The SD trigger acts on the sex-regulatory network by promoting the genetic pathway of one of both sexes, and/or by repressing the opposite sex^50–53^. To understand if *id2bbY* is directly promoting the male SD program or repressing the female pathway it is important to investigate the ancestral function of *id2b*. Arapaima *id2ba* and *id2bbY* have a male-specific expression in juvenile, but *id2bbY* is significantly higher expressed, also in adult testis. In both, juvenile and adults testis, TGF-β signaling genes are strongly expressed, consistent with a male-promoting role. In addition, the 3-D analyses of the Id2bbY protein demonstrates no notable difference in the HLH domain, indicating that it is functional and can have some overlapping role with Id2ba. Interestingly, expression analyses of the *id2* genes in sterlet and medaka showed higher expression in ovary compared to testis (Table S1), indicating that the male-bias of *id2a* and *id2b* of arapaima may be specific for this species.

To confirm the role of *id2bbY* as the master SD gene and its connection to TGF-β signaling, knock-out and gene addition genetic manipulations have to be done. Such experiments would also be useful to elucidate the role of Id2 proteins in sexual development. To date, however, the methodologies for genome modification and transgenesis are not developed in arapaima, yet.

In summary, we provide further evidence for the importance of TGF-β signaling in testis development, from which several upstream components are recurrently recruited as sex-determining trigger in different species. The *id2bbY* gene is the first candidate sex determining gene from the pathway that is located at a downstream position of the pathway. *id2bbY* is a reliable male -specific marker, which provides a versatile tool for sexing fish in aquaculture and for conservation measures.

## Material and methods

### Animals

Fish of *Arapaima gigas* (Schinz, 1822) used in this study were purchased from commercial farms in different regions of Brazil and from a population reared in Germany. In Brazil, the fish were from Cacoal (Rondônia), Coari (Amazonas), Presidente Figueiredo (Amazonas), Pimenta Bueno (Rondônia), Pentecoste (Ceará), Senador Guiomard (Acre) and Senador La Rocque (Maranhão). In Germany, gonads of 24 subadult arapaima were sampled during routine slaughtering at a commercial farm (Manich Food Innovations GmbH, MFI) in Thuringia, Germany (“Thuringia population”). Fish were imported from a farm in Colombia and reared for ~ 1 year in a recirculation aquaculture system (RAS) according to the companies’ production protocols. At time of slaughter, size of the sampled fish ranged from 94 to 1120 cm (average 106.6 cm) with a weight of 9 to 19.4 kg (average 13.2 kg).

For Hi-C, blood from one adult male (~ 6 years old) was taken during routine rearing procedures at the Leibniz-Institute of Freshwater Ecology and Inland Fisheries (IGB) in Berlin, Germany. Fish at IGB were imported from a farm in Peru and reared in groups at ~ 25 °C in recirculation aquaculture systems (RAS). Blood samples (1.5 ml) were taken with a heparinized syringe into 2 ml cryotubes, filled with 270 µl of DMSO (final concentration: 15% DMSO), gently mixed, tubes placed into a Nalgene Freezing container filled with isopropanol as recommended by the manufacturer and frozen within a container at − 80 °C for > 4 h until further processing.

### Genome sequencing and assembly

Genome sequencing and contig assemblies have been described in a previous paper^13^*.* Briefly, DNA was derived from fin tissue of a single adult female (ID F3) and from a single adult male (ID M14). Libraries were produced using the Truseq DNA Nano sample prep kit using the 550 pb insert size option and sequenced on a Hiseq 2500 using rapid v2 PE 2*250 nt mode. All sequences were assembled with *DISCOVARdenovo *(version 52488) (https://software.broadinstitute.org/software/discovar/blog/) using default parameters.

### Hi-C sequencing

In situ Hi-C was performed according to previously described protocols^54^. Cryopreserved blood cells were defrosted, washed with PBS twice and counted. 5 million cells were then cross-linked with 1% formaldehyde in PBS, quenched with glycine 0.125 M and washed twice with PBS. Membranes were then disrupted with a Dounce pestle, nuclei were permeabilized using 0.5% SDS and then digested with HindIII endonuclease. 5′-overhangs at *Hind*III-cut restriction sites were filled-in, in the presence of biotin-dCTP with the Klenow large fragment, and then re-ligated at a NheI restriction site. Nuclei were lysed and DNA was precipitated and then purified using Agencourt AMPure XP beads (Beckman Coulter) and quantified using the Qubit fluorometric quantification system (Thermo). T4 DNA polymerase was used to remove un-ligated biotinylated ends. Then, the Hi-C library was prepared according to Illumina’s protocols using the Illumina TruSeq Nano DNA HT Library Prep Kit with a few modifications: 1.4 μg DNA was fragmented to 550 nt by sonication. Sheared DNA was then sized (200–600 bp) using Agencourt AMPure XP beads, and biotinylated ligation junctions were captured using M280 Streptavidin Dynabeads (Thermo) and then purified using reagents from the Nextera Mate Pair Sample preparation kit (Illumina). Using the TruSeq nano DNA kit (Illumina), the 3′ ends of blunt fragments were adenylated. Next, adaptors and indexes were ligated, and the library was amplified for 10 cycles. Library quality was assessed by quantifying the proportion of DNA cut by endonuclease *Nhe*I using a Fragment Analyzer (Advanced Analytical Technologies, Inc., Iowa, USA). Finally, the library was quantified by qPCR using the Kapa Library Quantification Kit (Roche). Sequencing was performed on an Illumina HiSeq3000 apparatus (Illumina, California, USA) using paired-end 2 × 150 nt reads. This produced 78 million read pairs (23.5 Gb of raw nucleotides).

### Genome scaffolding

Contigs were scaffolded using Hi-C as a source of linking information. Reads were aligned to the draft genome using Juicer^55^ with default parameters. A candidate assembly was then generated with 3D de novo assembly (3D-DNA) pipeline^56^ with the − r 0 parameter. Finally, the candidate assembly was manually reviewed using Juicebox Assembly Tools^57^. Genome completeness was estimated using Benchmarking Universal Single-Copy Orthologs (BUSCO) v3.0^58^ based on 4584 BUSCO orthologs derived from the Actinopterygii lineage.

### Genome annotation

Repeats in the assembly were identified and masked by RepeatModeler and RepeatMasker (http://www.repeatmasker.org). The assembly was first scanned by RepeatModeler for de novo identification of repeats. The results, together with FishTEDB^59^, were then transferred to RepeatMasker for similarity scan and final repeat masking.

Protein coding genes were annotated by collecting and synergizing gene evidence from homolog alignment, expression data mapping and *ab inito* prediction^60^. Briefly, for homolog alignment, 611,738 protein sequences from Swiss-Prot (www.uniprot.org) and from related fish species were used as query. These species include *Paramormyrops kingsleyae*, *Erpetoichthys calabaricus*, *Scleropages formosus*, *Lepisosteus oculatus*, *Salmo salar*, *Danio rerio* and *Callorhinchus milii* (www.ensembl.org). GeneWise^61^ and Exonerate (https://www.ebi.ac.uk/about/vertebrate-genomics/software/exonerate) were used independently to align protein queries to the assembly and to determine the gene structure. GenBlastA^62^ were also used as support to GeneWise to roughly locate each protein sequence on the assembly. For expression data mapping, RNA-seq reads used in our previous study^13^ were mapped on the assembly using HISAT^63^ and parsed using StringTie^64^ for gene structure. In parallel, transcripts were first assembled using Trinity ^65^ and then mapped to the assembly using PASA^66^. For ab initio prediction, SNAP (https://github.com/KorfLab/SNAP), GeneMark-ES^67^ and AUGUSTUS^68^ were used.

Finally, gene structures that were consistently predicted in each of the above parallel approaches were selected as high-quality gene models to train AUGUSTUS, and all collected gene evidences were transferred to the trained AUGUSTUS for final annotation.

The final gene models were mapped to Pfam (https://pfam.xfam.org/), Swiss-Prot and NCBI nr database (fish only) using BLAST^69^, and genes with no hit and not supported by RNA-seq reads were removed.

### Identification of the male sex-specific marker by PoolSex

Pool-sequencing libraries were prepared from male and female gDNA pools of the same individuals analyzed in Du et al.^13^, using the Illumina TruSeq Nano DNA HT Library Prep Kit (Illumina, San Diego, CA, USA) according to the manufacturer’s protocol. After fragmentation of each gDNA pool (200 ng/pool) by sonication using an M220 Focused-ultrasonicator (COVARIS), the size selection was performed using SPB beads retaining fragments of 550 bp. Following the blunt 3′ end fragments mono-adenylation and ligation to specific paired-end adaptors, the amplification of the construction was performed using Illumina-specific primers. Library quality was verified with a Fragment Analyzer (Advanced Analytical Technologies) and then quantified by qPCR using the Kapa Library Quantification Kit (Roche Diagnostics Corp, Indianapolis, IN). The enriched male and female pool libraries were then sequenced using a paired-end multiplexed sequencing mode on a NovaSeq S4 lane (Illumina, San Diego, CA), combining both pools on the same lane and producing 2 × 150 nt reads with Illumina NovaSeq Reagent Kits according to the manufacturer’s instructions.

Pool-sequencing datasets were analyzed with the PSASS-workflow pipeline (https://github.com/SexGenomicsToolkit/PSASS-workflow) that computes on a whole-genome assembly, the fixation index (FS_T_), sex-specific single-nucleotide variation (SNVs, heterozygotes in one sex and homozygotes in the other sex), and male/female coverage differences. For the chromosome Manhattan plots, we used PSASS with the default settings i.e.,—window-size = 50,000,—output-resolution = 1000,—group-snps = True,—freq-het = 0.5,—range-het = 0.1,—freq-hom = 1, and—range-hom = 0.05. For the zoomed views of chromosomes 05 and 26, we used the small-window settings with a modified window-size = 5000,—output-resolution = 500.

Multiple alignment plots between the sex-biased region on chromosome 5 and the corresponding duplicated region on Chr26 of the male genome assembly were computed using mVista^70^ and the global pairwise alignment of finished sequences settings.

### Gene orthology assignment

Protein sequences of arowana (*Scleropages formosus*), coelacanth (*Latimeria chalumnae)*, fugu (*Takifugu rubripes*), spotted gar (*Lepisosteus oculatus*), Japanese medaka HdrR strain (*Oryzias latipes*), platyfish (*Xiphophorus maculatus*), reedfish (*Erpetoichthys calabaricus*), and zebrafish (*Danio rerio*) were downloaded from Ensembl and those of European eel (*Anguilla anguilla*) from NCBI. For each gene the longest protein sequence have been retained. We ran an all vs. all blast so each two genes received a similarity score (H-score^71^), based on which, Hcluster_sg^72^ clustered genes into different groups. For each group, a gene tree was built and then the orthology was finally assigned using TreeBesT (http://treesoft.sourceforge.net/treebest.shtml).

### Substitution rate analysis

Substitution rates were calculated using codeml in PAML^21^. Protein and coding sequences of *id2* were retrieved from NCBI and Ensembl, and aligned using ClustalW^73^ for protein sequence. Coding sequence alignments were obtained from the protein sequence alignment using PAL2NAL^74^. Phylogenetic trees were built based on the alignment using maximum likelihood method in MEGA7^75^.

### Genomic DNA extraction and genotyping

Muscle or caudal fins of arapaima were fixed in 100% ethanol and stored at 4 °C. The tissues were cut into smalls fragments and digested for 3 h at 60 °C in 750 µL extraction buffer (10 mM Tris, 0.1 mol/L EDTA, 0,5% SDS) and 10 µL proteinase K (20 mg/mL, Sigma-Aldrich). After digestion, 375 µL phenol and 350 µL chloroform:isoamyalcohol (24:1) were added and the phases mixed by gentle shaking for 10 min. After 12.000 rpm centrifugation at 4 °C the upper phase was transferred into a new tube, and 750 µL chloroform:isoamyalcohol (24:1) was added and mixed again for 10 min. The phases were separated by centrifugation. The genomic DNA contained in the upper phase was precipitated overnight at − 20 °C in 1400 µL absolute ethanol. After 12,000 rpm centrifugation the DNA pellet was washed twice with 70% ethanol and resuspended in 60 µL T.E. buffer (10 mM Tris–HCl and 1 mM EDTA). The genomic DNAs were quantified using NanoDrop-2000 and 100–250 ng was used in the PCR reactions.

The complete coding sequences of the male-specific gene of arapaima (*id2bbY*) and its autosomal copy (*id2ba*) were compared, and specific PCR primers (5′-CAAGTAGTCATTCAGAAACTTTTTCAG-3′ and 5′-GTACGTTGGATATAGATACACTTGGG-3′) for the Y chromosome copy were designed in the 3′UTR sequence of the gene (Fig. S5). The PCR products were resolved on 1% agarose gels.

#### Protein structure prediction

Protein sequence fasta files of arapaima Id2a, Id2ba and Id2bbY were submitted to the RaptorX protein structure distance based protein folding prediction site (http://raptorx.uchicago.edu/) and structures modelled by deep learning in both CASP12 and CASP 13^76–80^.

#### RNA sequencing and transcriptome analysis of juvenile and adult gonads

RNA of gonads of one juvenile male (RIN 9.0) and one juvenile female (RIN 7.0) arapaima were extracted using the TRIzol Reagent (Thermo Fisher Scientific, Waltham, USA) according to the supplier’s recommendation. RNA was then cleaned using RNeasy (Qiagen RNeasy Mini Kit cat#: 74104). Library processing and RNAseq were carried out by NOVOGENE (Cambridge, UK) on a NovaSeq 6000 PE 150, generating 10 Gb of data per sample.

Transcriptome sequences of male and female juvenile and adult gonads were mapped to the genome using the RNA-sequence aligner STAR (https://github.com/alexdobin/STAR/releases,—quantMode GeneCounts). Differentially expressed genes between testis and ovary were detected by DESeq2^81^ (Bioconductor/R) for juveniles and adults. Genes were considered to be differentially expressed, if *p* value ≤ 0.05 AND log2FC ≤ − 2 (higher expression in male) and log2FC ≥ 2 (higher expression in female). Heat maps for sex-related genes were plotted and genes showing comparable regulation between male and female, and between adult and juvenile samples were selected.

#### Sexing procedures

Gonads of 10 juvenile individuals (Thuringia population) were dissected from the body cavity prior to gutting of the fish. A central gonadal section of > 1 cm length was immediately transferred into 4% Histofix (Carl Roth, Germany) for histological analyses. All specimens from Coari, Pimenta Bueno, Presidente Figueiredo and Senador La Rocque were anaesthetized with 0.01% benzocaine (Acros, Morris, NJ, USA) and euthanized by cerebral concussion. A small sample of the fin was clipped for DNA extraction and a piece of the gonad was dissected and immediately fixed in 5% glutaraldehyde in phosphate buffer (0.1 mol l^−1^ at pH 7.2), dehydrated and embedded in glycol methacrylate (Leica, Heidelberg, Germany). Samples were cut in 5 μm thick sections and stained with hematoxylin–eosin staining as described^82^. Animals were sexed according to da Costa Amaral et al.^83^.

The animals originated from Pentecoste were sexed by endoscopy^84^ or vitellogenin detection, using the enzyme immune assay kit (Acobiom, Montpellier, France) developed specifically for *Arapaima gigas* according to the supplier’s recommendation.

#### Ethics approval and consent to participate

Adult and juvenile arapaima were kept and sampled in accordance with national German legislation. Rearing of Arapaima at IGB is according to authorization ZH 114 (issued 06 February 2014) by LAGeSo, Berlin, Germany. Commercial aquaculture at Manich Foods in Thuringia is authorized by registration 16 061 111S6001 (FischSeuchV). The maintenance, usage, and sampling of experimental arapaima in Brazil were approved by the Ethics Committee for the Use of Animals Embrapa Amazonia Ocidental, (protocols 04/2016, 01/2018 and 09/2020) accredited by the National Council for the Control of Animal Experimentation, which belongs to the Ministry of Science, Technology, Innovations and Communications. The study has Authorization of Access to Genetic Heritage from the Brazilian Ministry of the Environment (A5784B5 and ADE058A).

## Data availability

The raw data supporting the conclusions of this article will be made available by the authors, without undue reservation.

## Supplementary Information

Supplementary Figures.

Supplementary Table S1.
