# Supplementary material for: A duplicated copy of id2b is an unusual sex-determining candidate gene on the Y chromosome of arapaima (Arapaima gigas)
Source: Sci Rep. 2021 Nov 3;11:21544. doi: 10.1038/s41598-021-01066-z (PMC8566520; doi:10.1038/s41598-021-01066-z)
Supplement: Supplementary file 1 — Supplementary Figures. [file 41598_2021_1066_MOESM1_ESM.docx]

**Supplementary Figures**

**A duplicated copy of *id2b* in the male-specific region of the arapaima genome extends the spectrum of candidate sex-determining genes**

Mateus C. Adolfi^1*+^, Kang Du^1,2+^, Susanne Kneitz^3^, Cédric Cabau^4^, Margot Zahm^4^, Christophe Klopp^5^, Romain Feron^6,7^, Rômulo V. Paixão^8^, Eduardo S. Varela^9^, Fernanda L. de Almeida^8^, Marcos A. de Oliveira^10^, Rafael H. Nóbrega^10^, Céline Lopez-Roques^11^, Carole Iampietro^11^, Jérôme Lluch^11^, Werner Kloas^12^, Sven Wuertz^12^, Fabian Schaefer^12^, Matthias Stöck^12,13^, Yann Guiguen^6^, Manfred Schartl^1,2^

1 Developmental Biochemistry, Biocenter, University of Wuerzburg, Am Hubland, 97074, Wuerzburg, Germany

2 The Xiphophorus Genetic Stock Center, Department of Chemistry and Biochemistry, Texas State University, San Marcos, Texas, TX 78666, USA

3 Biochemistry and Cell Biology, Biocenter, University of Wuerzburg, Am Hubland, 97074 Wuerzburg, Germany

4 Sigenae, GenPhySE, Université de Toulouse, INRAE, ENVT, FR

5 MIAT, Université de Toulouse, INRA, Chemin de Borde Rouge, Castanet-Tolosan Cedex, F-31326, France.

6 INRAE, LPGP, Rennes, France

7 Department of Ecology and Evolution, University of Lausanne, and Swiss Institute of Bioinformatics, 1015, Lausanne, Switzerland

8 Embrapa Amazônia Ocidental, Manaus, Amazonas, Brazil

9 Embrapa Pesca e Aquicultura, Palmas, Tocantins, Brazil

10 Reproductive and Molecular Biology Group, Department of Morphology, Institute of Biosciences, UNESP, Botucatu, Brazil

11 GeT-PlaGe, INRAE, Genotoul, Castanet-Tolosan, France.

12 Leibniz-Institute of Freshwater Ecology and Inland Fisheries, IGB, Müggelseedamm 301 & 310, D-12587 Berlin, Germany

13 Amphibian Research Center, Hiroshima University, Higashi-Hiroshima 739-8526, Japan

* corresponding author: mateus.adolfi@biozentrum.uni-wuerzburg.de

+ These authors contributed equally to this work.

Figure S1


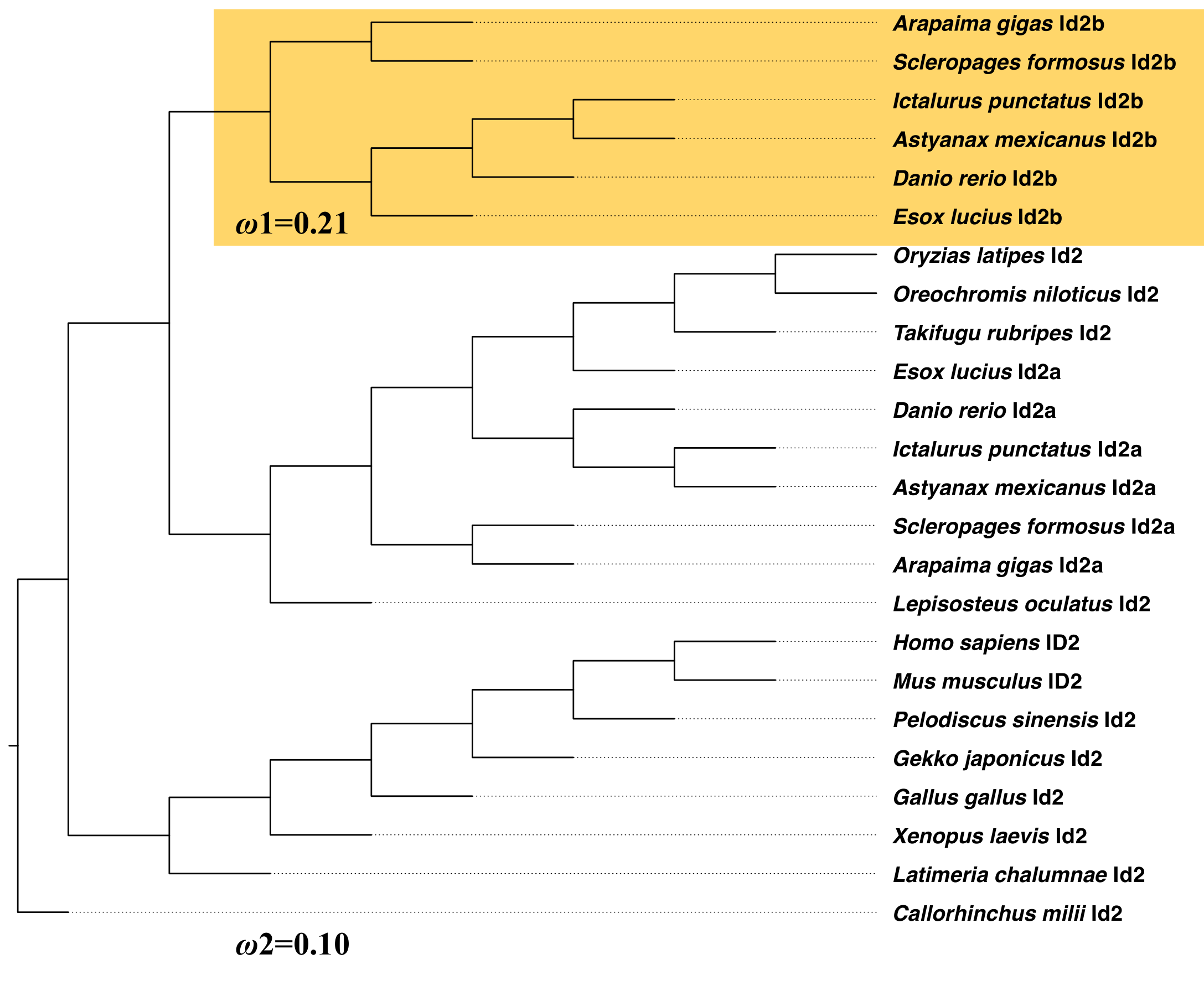


**Figure S1. Selection analysis of Id2a and Id2b.** Phylogeny plot showing arapaima Id2bbY (yellow) has a higher ω value (ω1=0.97) than Id2ba (ω2=0.25).

Figure S2


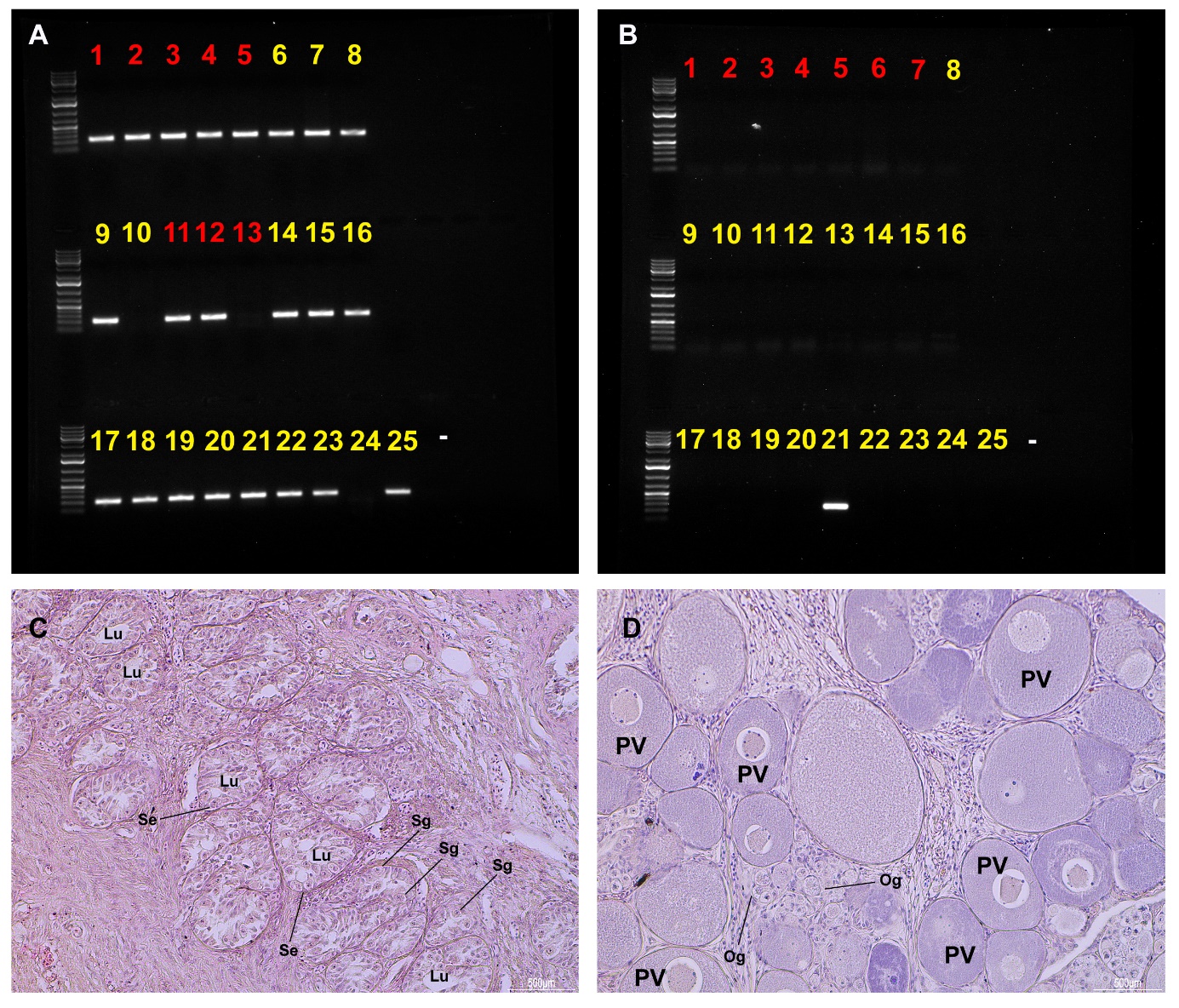


**Figure S2. Genotyping analyses of *id2bbY*.** Agarose gel electrophoresis gel resolution of the specific PCR amplification products of male (**A**) and female (**B**) used in the RAD-tag analyses from Senador Guiomard (yellow numbers) and Cacoal (red numbers) populations. Histological structure of juvenile testis (**C**) and ovary (**D**) from the samples used in the tests for genotyping. Lu. Lumen, Og. Oogonia, PV. Previtellogenic oocyte, Se. Sertoli cell, Sg. Spermatogonia.

Figure S3


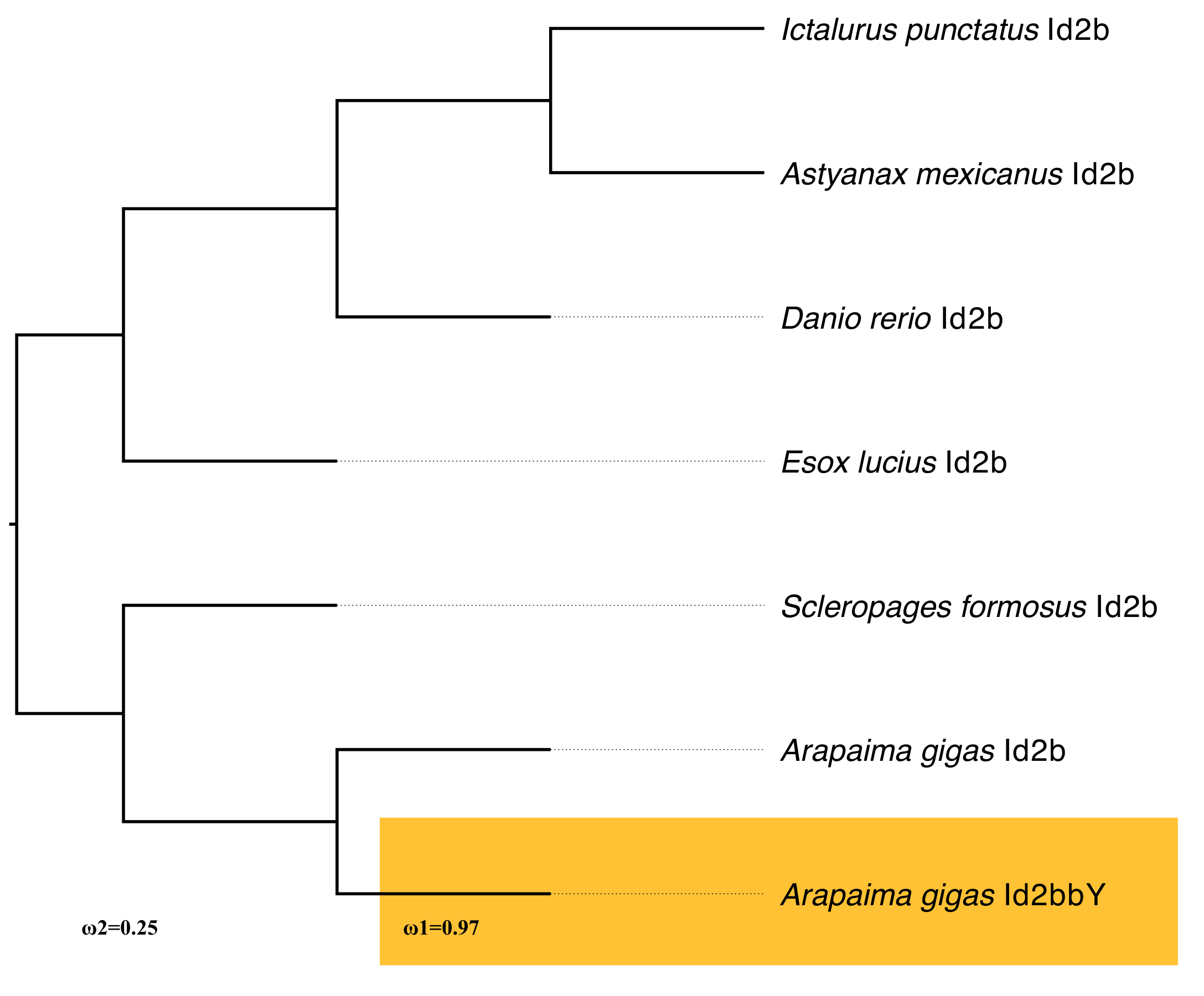


**Figure S3. Selection analysis of Id2bbY and Id2b of teleosts.** Phylogeny plot showing arapaima Id2bbY (yellow) has a higher ω value (ω1=0.97) than Id2ba(ω2=0.25).

Figure S4


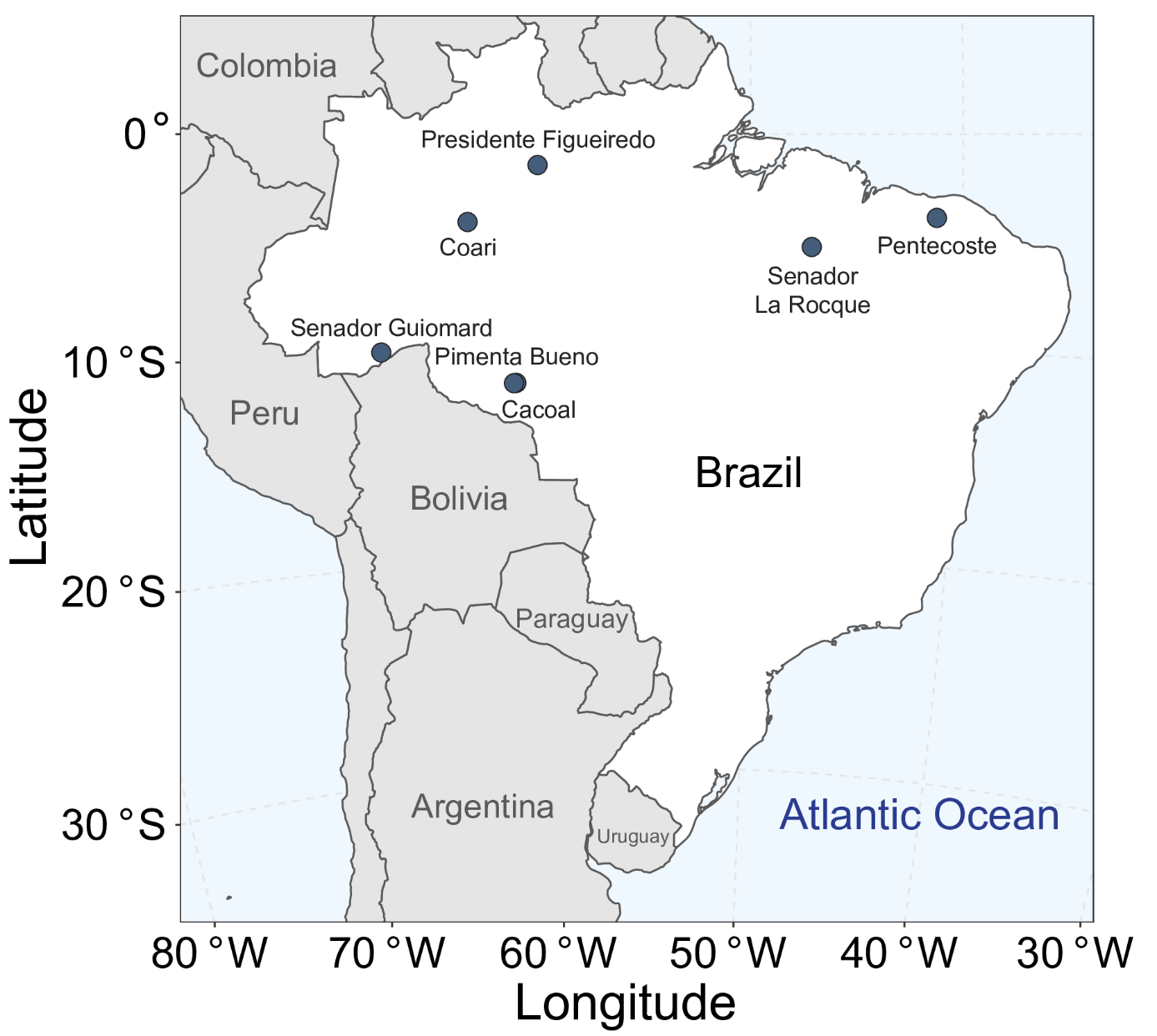


**Figure S4. Locations of the Brazilian arapaima populations used in the analyses.** Map was generated in R v4.0.2/RStudio v1.3.959 (RStudio Team, 2020) using R packages ‘ggplot2’ (v3.3.2) (Wickham, 2016) and ‘sf’ (v0.9-6) (Pebesma, 2018).

- Pebesma, E., 2018. Simple Features for R: Standardized Support for Spatial Vector Data. The R Journal 10 (1), 439-446, <https://doi.org/10.32614/RJ-2018-009>
- RStudio Team. (2020). RStudio: Integrated Development Environment for R. Boston, MA: RStudio, PBC.
- Wickham, H. (2016). ggplot2: Elegant Graphics for Data Analysis. Springer-Verlag New York.

Figure S5


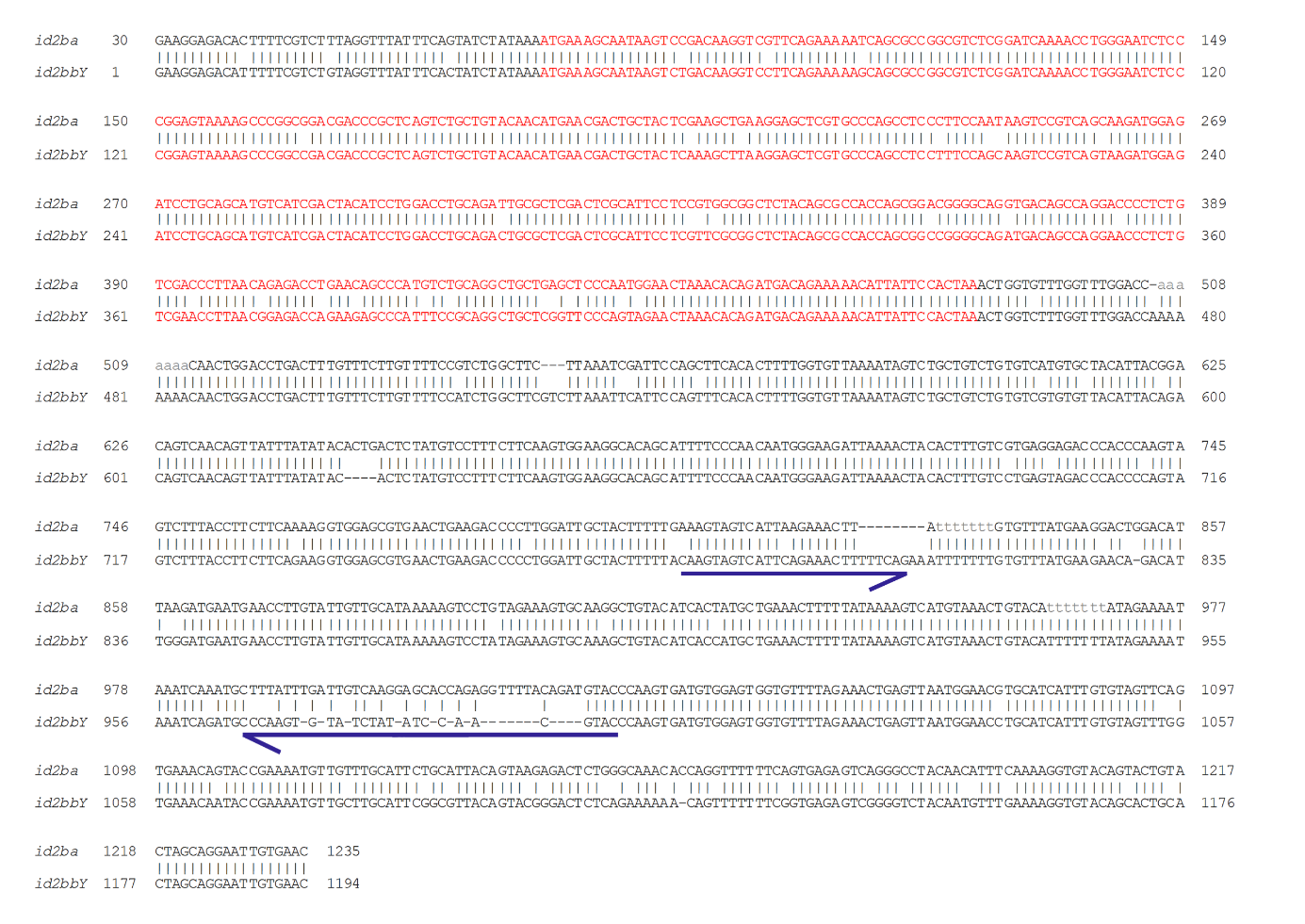


**Figure S5. cDNA sequence comparison between *id2ba* and *id2bbY*.** Nucleotide alignment of both *id2ba* and *id2bbY* showed high similarity in the open reding frame (red). The *id2bbY* specific primers (arrows) were designed in the 3’UTR region of the gene.
